# Metabolic Volume Measurements in Multiple Myeloma

**DOI:** 10.3390/metabo11120875

**Published:** 2021-12-16

**Authors:** Maria Emilia Seren Takahashi, Irene Lorand-Metze, Carmino Antonio de Souza, Claudio Tinoco Mesquita, Fernando Amorim Fernandes, José Barreto Campello Carvalheira, Celso Dario Ramos

**Affiliations:** 1 “Gleb Wataghin” Institute of Physics, University of Campinas (UNICAMP), Campinas 13083-859, Brazil; mseren@unicamp.br; 2 Department of Internal Medicine, Faculty of Medical Sciences, University of Campinas (UNICAMP), Campinas 13083-888, Brazil; ilmetze@unicamp.br; 3 Center of Hematology and Hemotherapy, University of Campinas (UNICAMP), Campinas 13083-878, Brazil; carmino@unicamp.br; 4 Departamento de Radiologia, Faculdade Medicina, Universidade Federal Fluminense (UFF), Niterói 24033-900, Brazil; claudiotinocomesquita@id.uff.br; 5 Hospital Universitário Antônio Pedro/EBSERH, Universidade Federal Fluminense (UFF), Niterói 24033-900, Brazil; fernando.fernandes2@gmail.com; 6 Division of Oncology, School of Medical Sciences, University of Campinas (UNICAMP), Campinas 13083-888, Brazil; jbcc@unicamp.br; 7 Division of Nuclear Medicine, School of Medical Sciences, University of Campinas (UNICAMP), Campinas 13083-888, Brazil

**Keywords:** multiple myeloma, 18F-fluorodeoxyglucose, positron emission tomography, metabolic tumor volume, total lesion glycolysis, intensity of bone involvement

## Abstract

Multiple myeloma (MM) accounts for 10–15% of all hematologic malignancies, as well as 20% of deaths related to hematologic malignant tumors, predominantly affecting bone and bone marrow. Positron emission tomography/computed tomography with 18F-fluorodeoxyglucose (FDG-PET/CT) is an important method to assess the tumor burden of these patients. It is often challenging to classify the extent of disease involvement in the PET scans for many of these patients because both focal and diffuse bone lesions may coexist, with varying degrees of FDG uptake. Different metrics involving volumetric parameters and texture features have been proposed to objectively assess these images. Here, we review some metabolic parameters that can be extracted from FDG-PET/CT images of MM patients, including technical aspects and predicting MM outcome impact. Metabolic tumor volume (MTV) and total lesion glycolysis (TLG) are volumetric parameters known to be independent predictors of MM outcome. However, they have not been adopted in clinical practice due to the lack of measuring standards. CT-based segmentation allows automated, and therefore reproducible, calculation of bone metabolic metrics in patients with MM, such as maximum, mean and standard deviation of the standardized uptake values (SUV) for the entire skeleton. Intensity of bone involvement (IBI) is a new parameter that also takes advantage of this approach with promising results. Other indirect parameters obtained from FDG-PET/CT images, such as visceral adipose tissue glucose uptake and subcutaneous adipose tissue radiodensity, may also be useful to evaluate the prognosis of MM patients. Furthermore, the use and quantification of new radiotracers can address different metabolic aspects of MM and may have important prognostic implications.

## 1. Introduction

Multiple myeloma (MM) is a hematological malignancy caused by the clonal expansion of plasma cells. It is one of the most frequent hematologic malignancies worldwide, accounting for 10–15% of them, as well as 20% of deaths related to hematologic neoplasms [1,2,3]. MM presents a heterogeneous distribution of tumor mass throughout the skeleton and may also exhibit extra-osseous foci.

Recently, new treatments have provided significant improvement in prognosis [4,5,6]. Therefore, it is critical to assess the whole tumor extension in the skeleton, as well as extra-osseous sites of involvement. Besides, it is necessary to monitor the evolution of the whole tumor mass during therapy [1].

Three-dimensional imaging techniques, such as whole-body computed tomography (CT), positron emission tomography/computed tomography (PET/CT) with 18F-fluorodeoxyglucose (FDG) and magnetic resonance imaging (MRI), are currently replacing whole body radiographs (WBR) for bone evaluation of these patients [7]. FDG-PET/CT detects the metabolic response early before morphological changes can be detected by conventional imaging or MRI [8]. Therefore, it is useful for assessing response to induction chemotherapy and autologous or allogeneic stem cell transplantation [8,9]. Furthermore, several studies have demonstrated the usefulness of FDG-PET/CT in determining prognosis, both at initial staging and at relapse or progression [10,11,12]. The increasing acceptance and use of FDG-PET/CT in the staging and monitoring of MM treatment response has led to the consensus statement issued by the International Myeloma Working Group (IMWG) in 2017 [13]. FDG-PET/CT was defined as the gold standard method to assess MM treatment response in several consensus publications [13].

FDG-PET/CT visual interpretation presently relies on semi-quantitative measures, such as the Deauville score, which was prospectively validated only for lymphomas [14,15]. The definition of FDG-PET positivity is currently defined by visual criteria that can be biased by inter-observer variability [12]. Recently, an attempt to better classify the amount of tumor mass was made by Nanni et al. [16,17] using an extensive stratified scoring system, which does not completely eliminate the subjectivity of interpretation.

Quantitative metrics are less operator-dependent than visual methods and can facilitate inter and multi-center clinical discussions [18]. Attempts have been made to standardize quantitative interpretations of FDG-PET/CT in MM, especially using parameters that refer to active metabolic disease [12,19,20,21,22]. However, the main limitation of FDG-PET/CT to measure the metabolic tumor volume in MM is to standardize the criteria for delimiting the areas affected by the disease. This impacts the reproducibility of interpretations, especially when assessing response to therapy. Therefore, there is a need for standardized and reproducible methods for quantifying the tumor burden using FDG-PET/CT.

Here, we review several previously reported quantitative parameters to assess FDG-PET/CT images, including standardized uptake value (SUV) and its derivatives, metabolic tumor volume (MTV), total lesion glycolysis (TLG), percentage of bone involvement (PBI) and intensity of bone involvement (IBI). Moreover, non-FDG radiotracers potentially quantifiable by these same techniques, and other tomographic imaging methods used for MM management, are briefly reviewed.

## 2. Methods to Quantify MM Tumor Burden Using FDG-PET/CT Images

Currently, the most used numerical metabolic metric for oncological PET is the SUV. In addition, volumetric parameters and texture features have also been promisingly discussed in the context of MM. The main metabolic metrics are discussed below.

### 2.1. SUV and Its Derivations

SUV is a dimensionless parameter, which is the activity concentration (*C*) within a region normalized by the injected activity (*A*) and patient weight (*P*) for a given time t (Equation (1)). If *C* is in units of mBq/mL, *P* should be used in grams (g) and *A* in mBq [23].

(1) $$SUV\left(t\right)=\frac{C\left(t\right)}{A\left(t\right)/P}$$

Maximum SUV (SUVmax) is the highest SUV value found in an area of interest, which can be a single lesion, a set of lesions or even the whole body. An SUVmax calculation of an attenuation-corrected PET image is simple, fast, reproducible and widely used in MM. High SUVmax values of FDG are related to high metabolic lesions, which in turn, may be related to a worse prognosis of MM patients. Stolzenburg et al. [19] related SUVmax values to worse overall survival (OS) both pre-treated and post-allogeneic hematopoietic cell transplantation (SUVmax > 6.54 and SUVmax > 2.81, respectively) for MM patients. Zamagni et al. [12] found that an SUVmax > 4.2 at baseline FDG-PET associated with at least three focal lesions and extramedullary disease decreases the progression-free survival (PFS) over 4 years in patients with newly diagnosed MM (NDMM). In addition, they found that SUVmax > 4.2 is an independent prognostic factor when it is still present after first-line treatment.

A prospective multi-center study evaluated SUVmax reduction (∆SUVmax) and known prognostic factors, such as the Revised International Staging System (R-ISS) and biochemical response, after three cycles of chemotherapy [24]. They conclude that ∆SUVmax is an independent prognostic factor and superior to visual analysis (Deauville 5-point score) in predicting PFS. Interestingly, R-ISS and biochemical response did not reach significance for PFS in the univariate analysis of this study.

Despite these good SUVmax results in MM, it presents known limitations [25,26,27,28,29]. SUVmax is based on a single image voxel and is very susceptible to several factors such as glucose blood level, body composition, size of the lesions, breathing movements and also image acquisition, reconstruction and correction methods [25,26,27,28,30].

**Figure 1:**
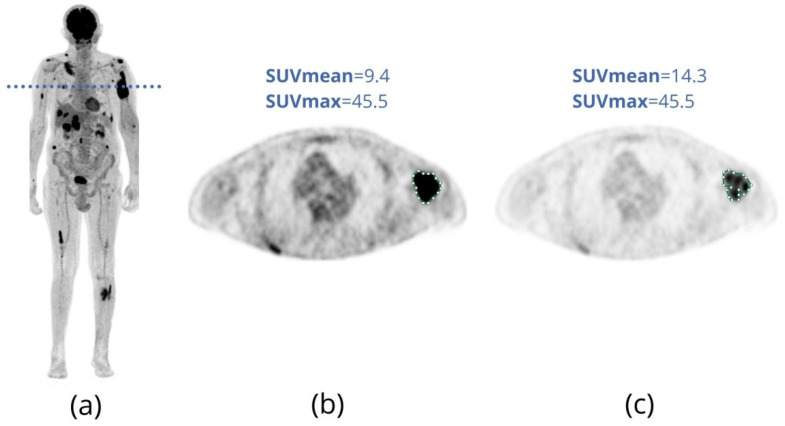
(**a**) FDG-PET/CT maximum intensity projection (MIP) image of an 85-year-old male patient with multiple myeloma and extensive bone involvement. The patient has several focal lesions spread throughout the body, the left humerus lesion being the one with the highest intensity of FDG uptake. (**b**,**c**) The same FDG-PET axial slice obtained at the level of the blue dotted line depicted in (**a**), but displayed with different image window settings. Different image windowing leads to different operator visual experiences, resulting in disparate contours for manual ROI (green lines). In (**b**), the lesion appears to be larger and with more uniform FDG uptake than in (**c**), resulting in different SUVmean values of 9.4 and 14.3. Note that while SUVmean is highly dependent on the ROI defined by the operator, SUVmax remains the same in both cases (45.5).

SUV derivations, such as SUVpeak, mean SUV (SUVmean) and standard deviation of SUV (SD_SUV_) are less affected by image noise than SUVmax since they are based on radiotracer uptake in a specific region, and outliers end up being softened. SUVmean is calculated as the arithmetic mean of SUV in a region of interest (ROI). The most common method to calculate SUVpeak uses the average SUV inside an ROI centered in the highest uptake volume of the lesion. However, there is a wide variation in the academic literature about how to define the area of highest uptake, size and format of the ROI [29,31]. Thus, both SUVmean and SUVpeak are highly affected by the determination of the area or volume of interest by the operator (Figure 1).

Amini et al. [32] retrospectively evaluated radiological and metabolic measurements at the level of L4 on FDG-PET/CT images performed at diagnosis of smoldering myeloma. SUVmax and SUVmean were measured using a circular ROI in the bone marrow. They found SUVmean as a prognostic indicator for PFS in these patients. None of the CT measures were associated with PFS [33].

Ak and Gulbas [33] used geometric ROIs to measure the mean SUVmax (mSUV) of the two femurs of 31 patients (21 with NDMM and 10 with relapsed disease after therapy). They found a strong positive correlation (*p* = 0.000, r = 0.755) with the percentage of myeloma cells expressing CD38/CD138 in bone marrow filtration of patients with MM. Furthermore, mSUV was also positively correlated with serum beta-2-microglobulin levels and negatively correlated with serum albumin levels.

**Figure 2:**
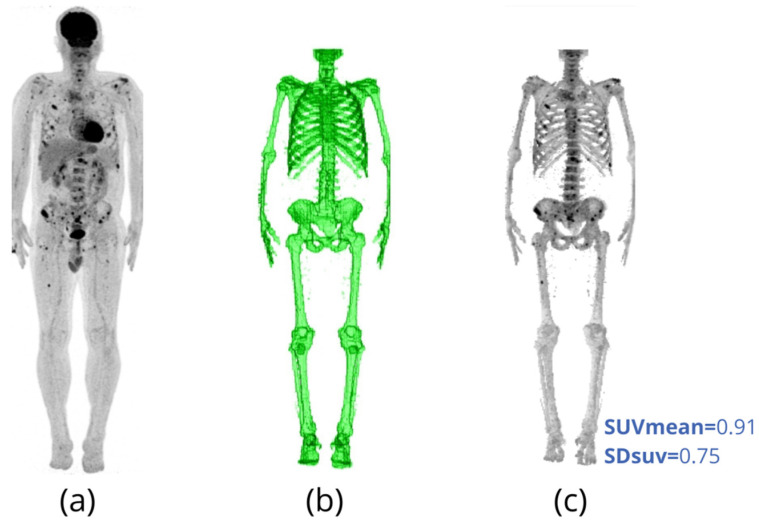
(**a**) Maximum intensity projection (MIP) image of the follow-up FDG-PET/CT of a 58-year-old male patient with multiple myeloma and mild bone involvement. (**b**) Bone segmentation mask obtained from CT images of the FDG-PET/CT shown in (**a**). The skull is excluded because of the intense overlap of cerebral FDG uptake on the cranium [34,37]. (**c**) MIP of FDG-PET images after bone segmentation using CT. Note that CT segmentation of the bone allows reproducible calculation of the mean SUV (SUVmean) and its respective standard deviation (SDsuv) exclusive for bone and bone marrow tissues of the entire skeleton (except the skull). For this patient, the skeletal SUVmean is 0.91, and its Sdsuv is 0.75.

Takahashi et al. [34] retrospectively assessed SUVmax, SUVmean and SD_SUV_ in the whole skeleton of 101 MM patients using CT-based segmentation. They found that both SUVmean and SD_SUV_ agree better than SUVmax with FDG-PET/CT visual analysis. Other studies have also used CT-based segmentation to assess changes in overall skeletal uptake to monitor MM outcome [35,36]. This method completely ignores hot spot volumetric data. However, it can provide a good overview of the overall state of skeletal tumor burden, including slight increases in baseline values (Figure 2).

### 2.2. MTV and TLG

MTV and TLG are volumetric parameters used to quantify the tumor burden of cancer patients. MTV is usually expressed in milliliters (mL) or cubic centimeters (cm^3^) [38]. MTV segmentation can be made manually, automatically or using a hybrid method (semi-automatic) [39]. Manual segmentation is not recommended for MM patients, as many lesions spread throughout the body are often present. Thus, manual segmentation becomes exhaustive and poorly reproducible [40].

Automatic or semi-automatic segmentation can be performed using thresholds or algorithm-based methods. Algorithm-based methods use logic, stochastic and learning-based techniques to obtain MTV [41]. Within this category are, for example, gradient-based methods, Gaussian mixture models, fuzzy locally adaptive Bayesian segmentation, Multi-Otsu method and those that involve machine learning [41]. These methods are not widely available on commercial workstations. This probably explains why they are not used very often for MM.

Threshold-based methods can use a fixed cut-off to determine the MTV (fixed threshold method) or can be based on specific image findings to determine an optimal threshold (iterative and adaptive methods) [40,41]. Fixed threshold is the most common method to calculate MTV. Fixed cut-off values can be based on a percentage of the SUVmax found in an individual image, or an intra-patient reference (e.g., mean liver uptake), or even an absolute global value, such as SUV = 2.5 [40].

TGL is derived from MTV. It is calculated by multiplying the MTV by its SUVmean. For this reason, in addition to the volumetric factor, TLG takes into account the intensity of radiotracer uptake [21].

Fonti et al. [20] calculated MTV and TLG in NDMM patients classified as stage IIIA according to the Durie and Salmon staging system. They used a semi-automatic method with a 40%-SUVmax threshold within a predetermined ROI with SUV > 2.5 to calculate MTV and TLG. MTV was positively correlated with the percentage of diffuse infiltration of bone marrow by plasma cells, and inversely correlated with hemoglobin levels. Furthermore, TLG was positively correlated with beta-2-microglobulin levels. A multivariate analysis of this study showed that MTV > 42.2 mL and MTV > 77.6 mL, were related to worse PFS and OS, respectively.

McDonald et al. [21] found that TLG > 620 g and MTV > 210 cm^3^ at baseline FDG-PET/CT are associated with worse PFS and OS in patients with MM. In this study, SUVmax and total number of focal lesions were not considered relevant as prognostic factors. The authors also investigated the relationship between TGL and 70-gene expression profiling (GEP) and International Staging System (ISS), which are known risk factors. Patients with low-risk GEP and high TLG had similar outcomes as high-risk GEP patients. The Chi-square test verified that the GEP and TLG are independent prognostic factors. Furthermore, they found that TLG > 205 g accurately divided ISS stage II patients into two subgroups with outcomes similar to those of ISS stage I and ISS stage III, respectively [22].

Terao et al. [22] utilized an absolute fixed threshold of SUV = 2.5 to calculate MTV and TLG from FDG-PET/CT images of NDMM patients. In a multivariate analysis, a high-burden MTV (>56.4 cm^3^) had prognostic values for PFS and OS even when the model was adjusted for the R-ISS and high-risk FDG-PET findings. The prognostic significance of PFS and OS was maintained when high-TLG (>166.4 g) was used in multivariate analysis instead of high-MTV. In both models, MTV and TLG had a higher hazard ratio (HR) than R-ISS for PFS (1.53 vs. 1.45 and 1.56 vs. 1.55, respectively), while R-ISS had higher HR than MTV and TLG for OS (2.65 vs. 2.10 and 2.72 vs. 2.19, respectively).

In a more recent study, Terao and collaborators [42] calculated tumor metabolic heterogeneity (MH) of lesions with the highest MTV and the highest SUV on FDG-PET/CT images of untreated NDMM patients. High MH calculated using the lesion with the highest SUV (MH-SUV) proved to be more important for survival analysis than using the highest MTV. MM patients with high MH-SUV had poorer PFS and OS than those with low MH-SUV. When MH-SUV and high-risk cytogenetic abnormalities (Cas) data were analyzed together, the patients with low MH-SUV and absence of high-risk Cas had better survival than those with high MH-SUV and presence of high-risk Cas, concomitant or not.

**Figure 3:**
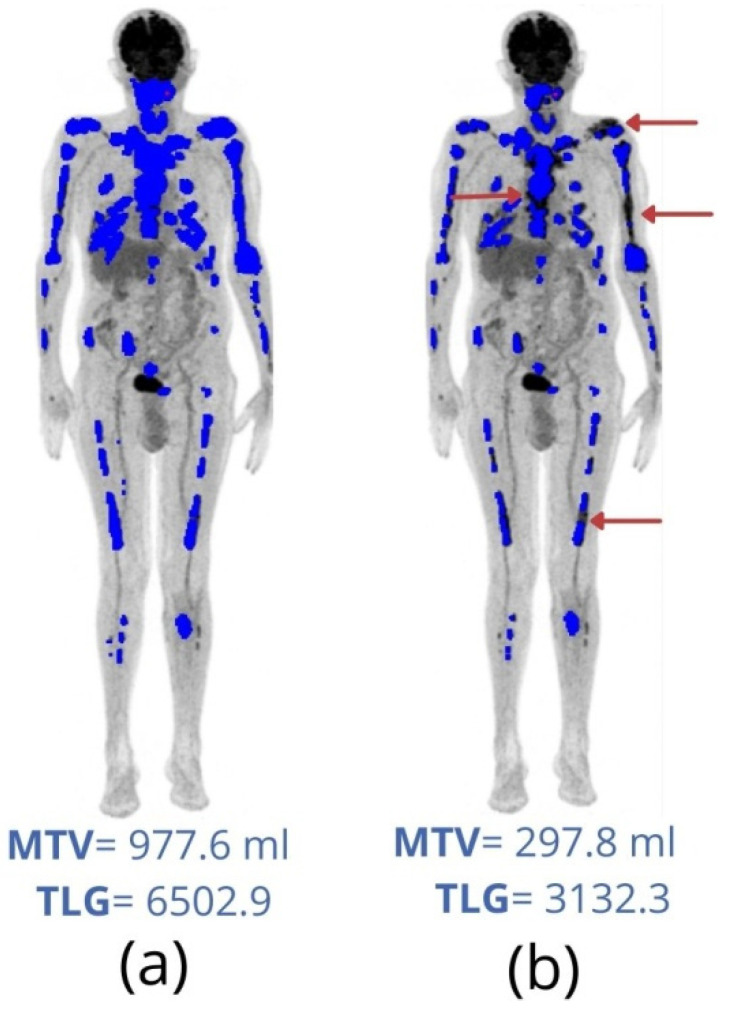
Metabolic tumor volume (MTV) and total lesion glycolysis (TLG) were calculated with two different fixed thresholds on the diagnostic FDG-PET/CT image of an 84 years-old male patient. Segmented volumes are highlighted in blue. (**a**) Using a fixed threshold of SUV = 2.5 results in an MTV = 977.6 mL and TLG = 6502.9. (**b**) Using a threshold of 41% of the maximum SUV (SUVmax) of each lesion, the resulting volumes were much lower: MTV = 297.8 mL and TLG = 3132.3. Note the individual lesions with lower volumes using 41% of SUVmax than SUV = 2.5 thresholds in this patient (arrows).

There is still no consensus on the best method for MTV calculation [41]. Furthermore, there is no consensus on the unit used for TLG, which could be presented as a dimensionless unit, a mass unit (grams) or a volumetric unit (ml or cm^3^) [38]. The European Association of Nuclear Medicine (EANM) guidelines for tumor imaging published in 2015 [43] recommends that, whenever possible, MTV and TLG calculated using the threshold of 41% or 50% of SUVmax should be reported. In a phantom study, 41% of isocurve appears to be the best representation of MTV [44]. However, when a low tumor-to-background ratio and/or non-homogenous uptake is found, other methods can be more suitable for MTV calculation [41]. For lymphoma, Eude et al. [45] found that MTV based on a fixed cut-off was significantly more reproducible than MTV based on 41% of the SUVmax. For MM patients, Li et al. [46] consider in their study that an FDG uptake superior to hepatic uptake is more appropriate to differentiate normal and pathological uptake in bone marrow. Considerable variations in MTV and TLG can be found when different thresholds are used (Figure 3).

### 2.3. PBI and IBI

**Figure 4:**
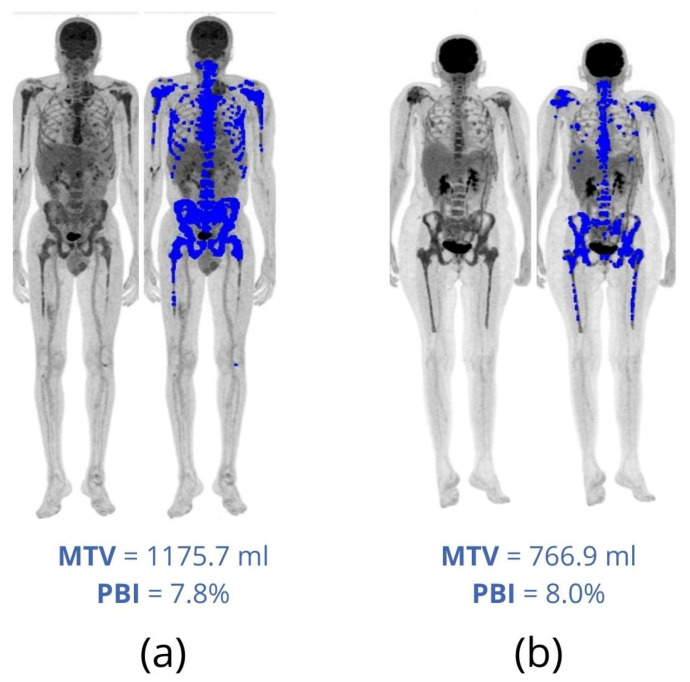
Diagnostic FDG-PET/CT of two different patients with MM. (**a**) A 61-year-old male, 1.75 m tall, weighing 71 kg. (**b**) A 47-year-old female, 1.52 m tall, weighing 58 kg. Involved areas with FDG uptake greater than liver uptake are highlighted in blue. Note that the patient in (**a**) presents higher metabolic tumor volume (MTV) but lower percentage of bone involvement (PBI) than the patient in (**b**).

PBI and IBI were proposed by Takahashi et al. [37] to quantify the total bone and bone marrow involvement in MM patients. A total segmentation of FDG uptake in bone tissue is needed to calculate PBI and IBI. For that, the authors used the Hounsfield scale of CT images to segment bone and bone marrow on the co-registered PET. After this step, PBI is calculated as the percentage of the total skeletal volume whose FDG uptake is higher than that of the liver, that is, the percentage of the total skeletal volume that is hypermetabolic. PBI is similar to an MTV normalized by the total skeletal volume of each patient. This normalization makes PBI less dependent on the patient’s height and gender. Patients with different sizes and equivalent MTVs do not have the same percentage of bone volume involved by the disease or vice versa (Figure 4).

IBI is calculated as PBI multiplied by its SUVmean. Thus, IBI also takes into account the intensity of radiotracer uptake by the lesions. If PBI is similar to MTV, then the IBI is similar to TLG normalized by the bone and bone marrow volume of the patient.

IBI has been shown to present a good relationship with the visual analysis of the images, allowing for an objective gradation of the disease among different patients. Furthermore, MM patients with more than 10 focal lesions had a significantly higher IBI score than groups of patients with up to 3 focal lesions or those with 4 to10 focal lesions [37]. High IBI scores measured at diagnostic FDG-PET were associated with high risk of death [47]. IBI variation (∆IBI) between two consecutive FDG-PET/CTs seems to be suitable to quantify image changes during patient follow-up [47].

This method routinely includes full skeletal segmentation, being very practical for patients with a high number of bone lesions. Furthermore, because IBI uses an absolute fixed threshold to the entire skeleton, it includes areas of diffuse bone marrow uptake. Other volumetric methods such as MTV/TLG usually consider only focal-type lesions. Diffuse FDG uptake in the bone marrow seems to play an important role in the prognostic evaluation of MM patients [48] and probably reflects plasma cell infiltration [46].

**Figure 5:**
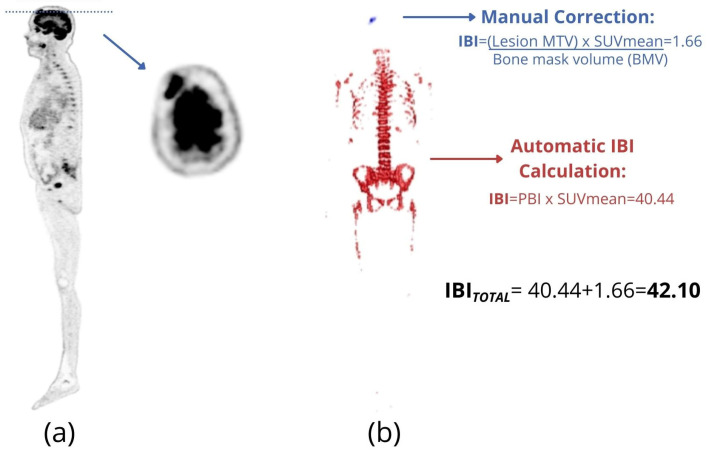
(**a**) Lateral maximum intensity projection (MIP) image (right) and axial slice at the level of the blue dotted line (left) of an FDG-PET/CT of a 67-year-old male patient with extensive bone involvement by multiple myeloma and a single focal lesion in the skull (arrow). (**b**) Volumes of interest (VOI) for IBI calculation. The volume highlighted in red was determined by CT-based segmentation, followed by the application of a fixed threshold based on the patient’s hepatic uptake (automatic segmentation) [37]. The skull lesion, highlighted in blue, was manually included in the IBI calculation.

Due to the low resolution of the PET image compared to CT image, areas of physiological uptake often overlap adjacent anatomical structures (partial volume effect). This effect is especially significant on the patient’s skull in FDG-PET/CT images. For this reason, when IBI is calculated on FDG-PET/CT images, the skull is excluded from CT-based segmentation. However, since some patients may have focal lesions in the skull, a manual correction for skull lesions inclusion would be necessary (Figure 5).

### 2.4. FDG Uptake of Adipose Tissue and Radiodensity

**Figure 6:**
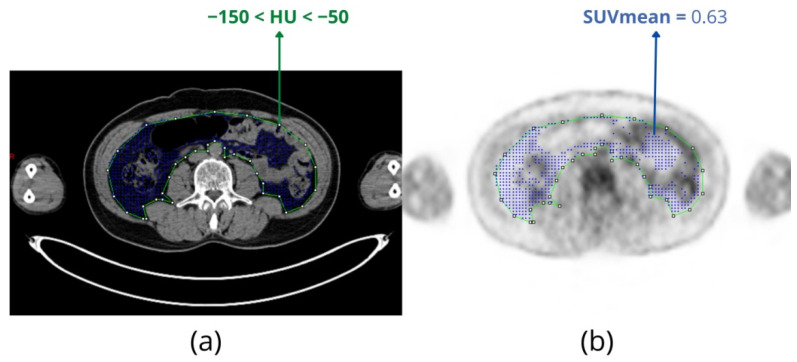
(**a**) A manual ROI (green line) was drawn on a CT axial slice at the level of L3 to enclose the visceral content. A threshold of −150 < HU < −50 was applied to segment the visceral adipose tissue (VAT), represented by the blue dots. (**b**) Anatomic region of VAT was then defined in the co-registered FDG-PET. SUV mean was calculated as the arithmetic mean of the SUVs of each pixel of segmented tissue corresponding to the blue dots.

Cachexia is present in more than 34% of patients with hematologic cancer and is associated with a respective increase in mortality of these patients [49]. In order to determine the pathophysiological features of cancer cachexia, accurate measurements of body composition are essential [50]. Cachexia is a complex process, and anthropomorphic measures such as body mass index (BMI) and skeletal muscle depletion may not be suitable as prognostic biomarkers for cancer survival [50,51,52]. Although “gold standard” techniques are still not available for precisely measuring cancer cachexia progression in patients with MM, emergent evidence indicates that abnormalities in adipose tissue depots are associated with survival outcomes in MM patients [53,54,55,56]. Specifically, increased visceral adipose tissue (VAT) was identified as a predictive factor of poor treatment response [53], while lower subcutaneous adipose tissue (SAT) was associated with reduced survival [54]. SAT radiodensity and increased VAT glucose uptake are associated with unfavorable prognosis in MM patients [55,56]. FDG uptake in VAT is usually measured in a slice at the third lumbar vertebra (L3) level. The segmentation criterion is the equivalent Hounsfield Unit (HU) for adipose tissue (Figure 6).

Cancer cachexia is a disorder characterized by wasting of muscle and adipose tissue, but changes in white adipose tissue (WAT) phenotype, i.e., the conversion of the white adipocytes in “beige” cells, was only described recently [57,58]. Interestingly, both high adipose tissue radiodensity and increased adipose tissue glucose uptake may be related to this phenomenon, and therefore, they may be early markers of cancer cachexia. In accordance, high SAT radiodensity has similar HU to that of brown adipose tissue [55,59,60]. This may also be a consequence of inflammation, which is also associated with high adipose tissue radiodensity. Increased FDG uptake in adipose tissue of MM patients can also be explained by adipocyte browning or by the presence of activated macrophages that are avid for glucose [61,62].

## 3. Artificial Intelligence for Estimating Total Metabolic Tumor Volume in Multiple Myeloma

Artificial intelligence (AI) can simulate intellectual work, and its use in nuclear medicine is becoming more and more relevant [63,64]. AI embraces executing tasks, such as understanding language and pattern recognition, recognizing objects and sounds or problem-solving. Machine learning is a part of AI and is related to the ability to learn from large amounts of data (a set of lessons) [65]. Recently, Yan et al. [66] demonstrated that machine learning models derived from routine laboratory results can accurately diagnose MM and can increase the rate of early diagnosis.

Deep learning is another important part of AI and refers to any neural network with more than one hidden layer (not a primarily input set of data). Hidden layers ultimately generate an output layer, which can perform standard classification/regression tasks. Convolutional neural networks (CNN) can work with visual data and process imaging information in much more detail than human capacity. CNN-based approaches have been demonstrated to effectively diagnose MM based exclusively on mass spectrometry data from peripheral blood [67]. Deep learning algorithms can use the full set of imaging data directly from the raw images in contrast to conventional machine learning approaches that require manual extraction of these features, a process that is tedious and may not fully capture the underlying imaging information for the task with potential selection bias [66].

Total tumor burden evaluation is not easily implemented in clinical practice because the exact identification and segmentation of each tumoral lesion to measure these indexes is time-consuming and is sometimes very challenging (multiple and disseminated lesions). Recently, Capobianco et al. [68] used CNNs to localize and classify uptake patterns of whole-body FDG-PET/CT images in patients with lymphoma and showed that this approach considerably simplified MTV estimation, reduced observer variability and facilitated the use of MTV as a predictive factor in lymphoma patients. The use of AI in PET images of patients with MM is still in its first steps. Morvan et al. [69] demonstrated that random survival forest radiomics analysis in MM patients reduced the errors of the predicted progression, increasing the predictive value of FDG-PET/CT in this scenario. Future studies are necessary to demonstrate in MM patients whether Al will be used as an aide for the interpreting physician by removing tedious and repetitive tasks of identification and classification of all lesions suspicious for malignancy while providing potentially accurate measurement of whole-body tumor burden [70].

## 4. Other Radiotracers Used for Multiple Myeloma

Most of the molecular imaging data currently available in MM is based on studies using FDG, a glucose analog. Therefore, all quantitative methods used to evaluate FDG-PET/CT are, of course, exclusively quantifying glucose metabolism. However, several other radiopharmaceuticals have been proposed to study MM.

**Figure 7:**
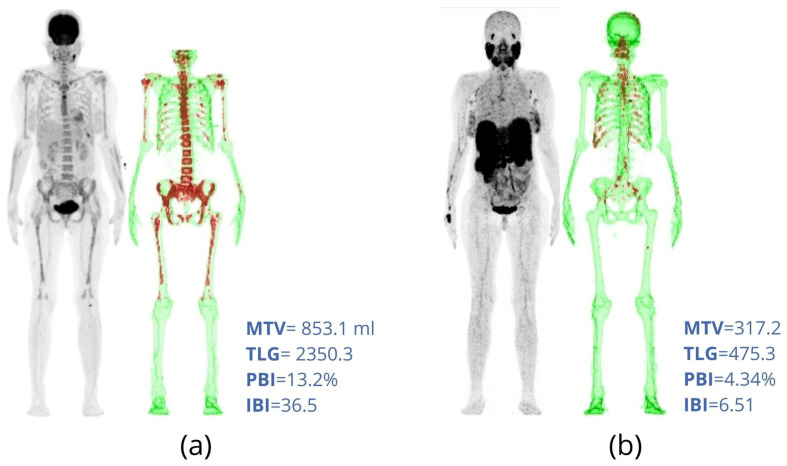
FDG-PET/CT (**a**) and 68Ga-PSMA PET/CT (**b**) of a 47-year-old female patient with bone involvement by multiple myeloma. The time interval between the exams was three days. The volumes of interest (VOIs) used for bone segmentation are highlighted in green. For FDG-PET/CT, the fixed threshold used for lesion segmentation was the mean hepatic uptake plus two standard deviations (SUV = 2.00). For PSMA-PET/CT, it was the mean uptake of the left atrium (blood pool) plus two standard deviations (SUV = 1.08). The bone and bone marrow areas in which radiotracer uptake is above the fixed threshold are highlighted in red. The quantitative parameters MTV, TLG, PBI and IBI are shown on the right of the images. Note that diffuse FDG uptake in bone tissue (**a**) was not detected by PSMA examination (**b**). However, a higher number of rib lesions were detected by PSMA (**b**) than by FDG (**a**) image. Moreover, note that it is not necessary to exclude the skull region for automatic contour of the VOI in the bone tissue for PSMA-PET/CT (**b**) because there is no physiological uptake of this tracer in the brain.

Single-photon emission computed tomography (SPECT)/CT using 99mTc-sestamibi (MIBI)—which is related to mitochondrial activity—has been demonstrated to be more efficient than FDG-PET/CT for detecting the diffuse involvement of bone marrow in MM [71]. 68Ga-labeled prostate-specific membrane antigen (PSMA) PET/CT can also detect MM lesions, probably by evidencing neoangiogenesis in the lesions [72,73] (Figure 7).

68Ga-Pentixafor is a new PET tracer with a high affinity for the chemokine receptor-4 (CXCR4), which is highly expressed in several hematologic malignancies. It seems to have greater positivity than FDG in MM [74]. Old PET tracers such as 11C-choline and 11C-methionine have also been used to study MM [75]. Choline is a component of phosphatidylcholine; therefore, 11C-choline is an indicator of plasma membrane synthesis. The radiolabeled amino acid methionine is supposed to be rapidly incorporated into newly synthesized immunoglobulins in MM lesions [75].

3′-Deoxy-3′-[18F]-fluorothymidine (FLT) is used to image DNA synthesis and indirectly evaluate cell proliferation. FLT-PET/CT has been proposed as a possible adjunct in the prognostic evaluation of MM patients. Finally, PET/CT using a somatostatin receptor expression marker—68Ga-DOTATATE—has also been shown to detect MM with an efficacy similar to that of FDG-PET/CT in a lesion-based analysis [76].

CD38 is a glycoprotein highly specific for MM, and anti-CD38 antibodies (e.g., daratumumab) have been successfully used to treat the disease [77]. Currently, CD38-based radiotracers for PET/CT imaging are also under evaluation [78,79,80]. The first human images of ^89^Zr or ^64^Cu-labeled anti-CD38 have recently been obtained, with promising results [78,79]. However, the low tumor-to-background ratio (TBR) resulting from full-size antibodies implies the need for premedication with unlabeled antibodies and delayed imaging, up to days after tracer injection [78,79]. More recently, ^68^Ga-labeled anti-CD38 single-domain antibodies have been used to obtain same-day pre-clinical images with high TBR [80].

It is interesting and intriguing that radiopharmaceuticals that represent such diverse metabolic aspects can identify the same disease. Generally, different tracers present different uptake intensities in different lesions of the same MM patient [71,74,75]. The entirely distinct uptake mechanisms of these radiopharmaceuticals suggest that this is related to the very heterogeneous biological behavior of this disease. Cytogenetic analyses have shown that MM is not a single disease, but has unique characteristics at the molecular level in each patient [81]. Therefore, the same volumetric quantitative methods described here for FDG-PET/CT can potentially be used for several other radiopharmaceuticals. Comparison of metabolic volumes of various tracers that represent different metabolic aspects of the disease could have clinical and prognostic implications. Possibly, this would contribute to the understanding of the already demonstrated expressive inter-patient and intra-lesion heterogeneity of MM [82].

## 5. FDG-PET in Comparison with MRI and CT

Imaging techniques are essential in the diagnosis and follow-up of patients with MM. They allow the assessment of the effects of the disease on the skeletal system and identify the presence of extramedullary disease. In the past, this included skeletal radiographic examinations, now largely replaced by CT, MRI and FDG-PET/CT [83,84,85,86,87].

While CT exclusively provides anatomical details of lesions, MRI can provide both anatomical and functional information, depending on the technique used. Since FDG-PET identifies metabolic alterations, it can even detect lesions without macroscopically detectable anatomical alterations. Particularly, hybrid PET/CT images bring together both metabolic and anatomical information. For this reason, FDG PET/CT can distinguish metabolic activity from inactive or necrotic lesions [88]. Therefore, it offers the additional advantage of assessing the degree of metabolic activity associated with myeloma lesions, while allowing adequate morphological characterization and assessment of potential complications such as pathological fractures [13]. The overall sensitivity for bone lesions varies from 80% to 100% at initial staging [89,90]. The method detects additional lesions in 24–50% of patients when compared to WBR [91]. Furthermore, it is especially helpful for identifying extramedullary disease [92], which presents a worse prognosis. A systematic review comparing conventional whole-body MRI with FDG-PET reported greater sensitivity, but lower specificity of MRI as compared to FDG-PET [93].

In the study conducted by Zamagni et al. [90], the authors compared FDG-PET/CT images with T1-weighted MRI of 46 MM patients at baseline. They conclude that MRI is superior in assessing spinal and pelvic bone marrow involvement. FDG-PET provided additional and valuable information for the assessment of MM bone disease in areas not covered by the MRI’s field-of-view (FOV). In this study, the ability to detect active sites of MM, both spinal and extramedullary, reached 92% when combining the spinal-pelvis MRI with whole-body FDG-PET/CT images. Other studies also support the concept that MRI and FDG-PET/CT are complementary techniques. [7,86,94,95,96,97].

Diffusion-weighted imaging (DWI) is a functional MRI imaging technique that reflects the rate of water diffusion between tissues. DWI is regarded as the most sensitive imaging technique for bone marrow lesions [87,97]. In parallel with FDG-PET, DWI allows quantitative assessments, such as measures related to the apparent diffusion coefficient (ADC) and volumetric measures such as total diffusion volume (tDV). Terao et al. [95] compared the prognostic significance of TLG and tDV for NDMM patients at baseline. They reported that although both tDV and TLG provided prognostic information about PFS, only TLG remained a prognostic factor for OS in the analyzed group. Interestingly, some patients present contradictory tDV and TLG, probably due to genomic heterogeneity. The genomic heterogeneity of MM lesions—including intra-patient lesions—is well-known [82]. For example, lesions that are FDG non-avid, and therefore undetectable by FDG-PET, are associated with low expression of hexokinase-2 [98,99]. These lesions can potentially be detected by MRI or PET with non-FDG radiopharmaceuticals.

Multi-center studies without methodological bias comparing MRI and FDG-PET/CT images in MM are lacking [93]. The roles of PET, MRI and CT in MM will certainly be re-discussed with the publication of new comparative studies and with the improvement of volumetric quantification techniques.

## 6. Conclusions

MM is still not a curable disease, and accurate monitoring of tumor mass is essential for disease management. FDG-PET/CT is an established method to assess tumor burden in these patients. Different metrics have been proposed to objectively assess the images, including MTV and TLG, which present challenges in standardization. PIB and IBI are new parameters based on reproducible CT segmentation of PET images, with promising results. Indirect parameters, such as FDG uptake in VAT may also be useful to evaluate the prognosis of these patients. AI, including deep learning and CNN, can simplify PET/CT quantifications and reduce observer variability. The quantitative methods currently used to evaluate PET/CT in MM are based on FDG images. The use of these methods to quantify images of tracers with different uptake mechanisms might contribute to the understanding of the expressive heterogeneity of the disease.

## Data Availability

Not applicable.
